# Predictive factors for distant metastasis in early cutaneous melanoma: A 20-year experience of a Turkish tertiary referral hospital^؆^

**DOI:** 10.1016/j.abd.2025.501212

**Published:** 2025-10-27

**Authors:** Ozlem Ozbagcivan, Elif Kazaz

**Affiliations:** Department of Dermatology, Dokuz Eylul University, Faculty of Medicine, Izmir, Turkey

**Keywords:** Aged, Geography, Melanoma, Cutaneous malignant, Neoplasm metastasis, Prognosis

## Abstract

**Background:**

Melanoma is an aggressive skin cancer with a high metastatic potential and mortality risk. Identifying risk factors for distant metastasis is crucial for optimizing surveillance strategies. The authors aimed to evaluate the predictors of distant metastasis in early-stage cutaneous melanoma within a Turkish patient cohort.

**Methods:**

A retrospective cohort study was conducted on patients with stage I–II melanoma, diagnosed between 2004 and 2024 at the Department of Dermatology, Dokuz Eylul University. A minimum follow-up period of five years was ensured. Demographic, clinical, and histopathological variables were analyzed for their association with distant metastasis using univariate and multivariate statistical models.

**Results:**

Among 148 patients, distant metastasis occurred in 36 (24.3%) during follow-up. Multivariate analysis identified age (HR = 1.03 per year, p = 0.050), Breslow thickness (HR = 1.19 per mm, p = 0.008), ulceration (HR = 3.05, p = 0.028), and Lymphovascular Invasion (LVI) (HR = 4.20, p = 0.002) as significant independent predictors of distant metastasis. The risk was found to increase markedly, particularly after the age of 40. Additionally, the optimal cut-off value for Breslow thickness was determined to be 2.73 mm, with a sensitivity of 69% and a specificity of 71% based on ROC curve analysis.

**Conclusion:**

Increasing age, Breslow thickness, ulceration, and LVI were identified as independent risk factors for distant metastasis in early-stage cutaneous melanoma. Notably, patients over 40-years and those with Breslow thickness >2.73 mm were at significantly higher risk. Further studies are warranted to validate these results and facilitate their integration into clinical practice.

## Introduction

Cutaneous melanoma is one of the most aggressive skin cancers, characterized by its high metastatic potential and significant mortality risk. Despite advancements in early detection and treatment, distant metastasis remains a critical factor influencing prognosis, often leading to poor survival outcomes. Identifying these risk factors is crucial for improving patient stratification, optimizing surveillance strategies, and developing targeted therapeutic approaches to mitigate the devastating impact of metastatic disease.^1^

Current guidelines established by the American Joint Committee on Cancer (AJCC) emphasize the significance of Breslow thickness as the most important prognostic factor for the primary tumor, with ulceration status also serving as an independent prognostic factor.^2^ However, investigating additional criteria that may predict patient prognosis has become increasingly important with recent advancements in melanoma treatment. Recent studies have demonstrated that several parameters, such as patient age, sex, anatomical location of the tumor, histological subtype, and specific histopathological features, are closely associated with distant metastasis and overall survival (OS).^3^, ^4^, ^5^, ^6^, ^7^, ^8^, ^9^ Nevertheless, the heterogeneity in study results in the literature emphasizes that the risk of distant metastasis in melanoma varies by geographic region, ethnicity, study design, and patient populations, highlighting the need for population-specific risk assessments and prognostic models.

There is a limited number of clinical and prognostic outcome studies reported from Turkey, and, to date, no long-term follow-up study has specifically addressed early-stage melanoma patients. This study aims to investigate clinical, demographic, and histopathological factors associated with distant metastasis in a cohort of Turkish patients with early-stage cutaneous melanoma. The findings are expected to provide novel insights and contribute to developing personalized management strategies for this population.

## Methods

### Ethics

The study received approval from the Local Ethics Committee and adhered to the guidelines outlined in the Declaration of Helsinki (Reference Number: 2025/03-05, Date: 22.01.2025).

### Study population

**Fig. 1:**
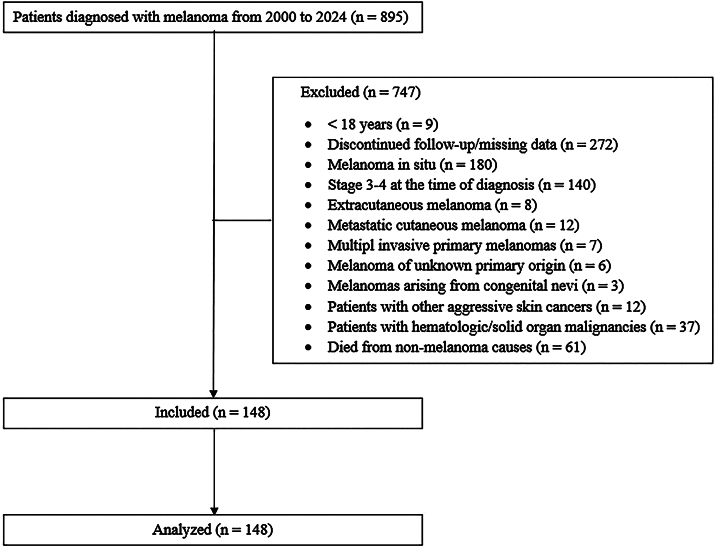
STROBE diagram for the retrospective cohort study conducted between 2000 and 2024.

The authors conducted a retrospective cohort analysis of cutaneous melanoma patients aged ≥ 18 years whose data were entered into the hospital registry system between 2004 and 2024 at the Department of Dermatology, Faculty of Medicine, Dokuz Eylul University. The sample size was determined by including all patients who met the inclusion criteria over a 20-year period at the studied institution, in order to enhance representativeness and avoid selection bias. The most recent diagnoses were made in 2019, ensuring a minimum follow-up period of five years for all surviving participants; however, patients who died due to melanoma within this five-year period were also included in the study. Patients were excluded if they had discontinued follow-up, died from non-melanoma causes, had a history of hematologic or solid organ malignancies, or met any of the following criteria: melanoma in situ, multiple invasive primary melanomas, other aggressive skin cancers with the potential for distant metastasis (e.g., squamous cell carcinoma, Merkel cell carcinoma), extracutaneous melanoma, melanoma of unknown primary origin, metastatic cutaneous melanomas, melanomas arising from congenital nevi, or clinical or microscopic evidence of Lymph Node (LN) or distant metastases at the time of diagnosis. Following these criteria, the final cohort consisted of patients with Stage I to II melanoma classified by the AJCC 8th edition (Fig. 1).

### Data collection

Demographic and clinical data, including age, gender, tumor location, radiological imaging results, and sentinel LN biopsy (SLNB) outcomes, were extracted from hospital records. Histopathology reports collected data on Breslow thickness (in millimeters), mitotic rate (in numbers), and presence of ulceration, regression, lymphovascular Invasion (LVI), and tumor-infiltrating lymphocytes (TIL). Breslow thickness values were rounded as per AJCC 8th edition recommendations. The primary outcome was identifying prognostic factors for distant metastasis (Stage IV), while the secondary outcome focused on locoregional cutaneous or LN metastasis (Stage III).

### Statistical analysis

Statistical analyses were performed using SPSS software (Version 24.0, SPSS, Inc., Chicago, Illinois). Missing or ambiguous data were categorized as “unknown”. Data normality was assessed using the Shapiro-Wilk test. Numerical variables were summarized as medians with interquartile ranges (IQR), while categorical variables were compared using χ^2^ or Fisher's exact tests in 2 × 2 tables when expected frequencies were < 5. The Mann-Whitney *U*-test was used for nonparametric comparisons between groups. Survival analysis was conducted using Kaplan-Meier curves and the log-rank test to compare survival curves, censoring patients who did not experience the event of interest. Univariate Cox proportional hazards regression analysis was used to evaluate the effect of individual variables on survival. Variables found significant in univariate analysis were included in a multivariate Cox regression model to calculate Hazard Ratios (HRs), 95% Confidence Intervals (95% CIs), and p-values. Receiver operating characteristic (ROC) curve analysis was performed to assess prediction accuracy. A p-value < 0.05 was considered statistically significant, with adjusted p-values applied in log-rank tests as necessary.

## Results

### Patient demographics

The study cohort comprised 148 Caucasian patients, including 101 females with a median age of 48 (range: 19–83, IQR: 31–65 years) and 75 males with a median age of 57 (range: 18–86; IQR: 43–68 years). A statistically significant age difference was observed between genders (p = 0.043). Table 1 presents a detailed summary of clinical and demographic data.

**Table 1:** Clinical, demographic, and histopathological characteristics of patients at the time of diagnosis.

Parameter	n	%
**Sex**		
Female	73	49.3
Male	75	50.7
**Age (years), median (quartiles)**		3.00 (35.25–66.00)
< 40	44	29.7
40‒60	49	33.1
> 60	55	37.2
**Melanoma subtype**		
SSM	83	56.1
NM	37	25.0
ALM	17	11.5
LMM	11	7.4
**Localization**		
Head and Neck	36	24.3
Trunk	51	34.5
Extremity	42	28.4
Acral	19	12.8
**Stage**		
Ia	49	33.1
Ib	18	12.2
IIa	28	18.9
IIb	21	14.2
IIc	32	21.6
**Sentinel Lymph Node Biopsy**		
Present	118	79.7
Absent	30	20.3
**Breslow (mm), median (quartiles)**		1.87 (0.72–4.00)
≤ 1	49	33.1
> 1‒2	26	17.6
> 2‒4	37	25.0
> 4	36	24.3
**Ulceration**		
Present	65	43.9
Absent	83	56.1
**Mitosis rate, median (quartiles)**		3.00 (1.00–11.00)
0	24	16.2
1‒5	58	39.2
> 5	50	33.8
Unknown	16	10.8
**LVI**		
Present	19	12.8
Absent	107	72.3
Unknown	22	14.9
**Regression**		
Present	44	29.7
Absent	81	54.7
Unknown	23	15.5

SSM, Superficial Spreading Melanoma; NM, Nodular Melanoma; ALM, Acral Lentiginous Melanoma; LMM, Lentigo Maligna Melanoma; LVI, Lymphovascular Invasion; TIL, Tumor-Infiltrating Lymphocytes.

### Rates and patterns of metastasis development in patients

During a median follow-up period of 7.0 years (IQR: 3–11 years), distant metastasis occurred in 36 out of 148 (24.3%) patients. Among these, 15 patients (10.1%) developed hematogenous metastasis only, 18 patients (12.2%) initially developed LN metastasis followed by hematogenous metastasis, and three patients (2.0%) experienced hematogenous metastasis before developing LN metastasis. 22 patients (14.9%) developed only locoregional cutaneous or LN metastasis (Table 2). The study cohort's 1-, 2-, and 5-year survival rates were 95.9%, 90.5%, and 60.8%, respectively.

**Table 2:** Metastasis distribution in melanoma patients at the end of the study.

Parameter	n	%
Stage III	22	14.9
Stage IV	36	24.3
**M-stage**		
M1a	2	5.6
M1b	8	22.2
M1c	17	47.2
M1d	9	25.0

### Association of distant metastasis with clinical and demographic data

Distant metastasis was significantly more frequent in stage 2b and 2c melanomas compared to earlier stages (p < 0.001, HR = 12.18 [4.94–30.04]). In multivariate analyses, the only demographic factor significantly associated with distant metastasis was increasing age (p = 0.050, HR = 1.03 [1.00–1.05]). The categorical analysis of age groups also revealed significant differences (p = 0.032), indicating that this difference was due to an increased risk of metastasis in individuals over 40-years of age compared to younger patients (adjusted p = 0.006, HR = 3.57 [1.38–9.24]).

**Fig. 2:**
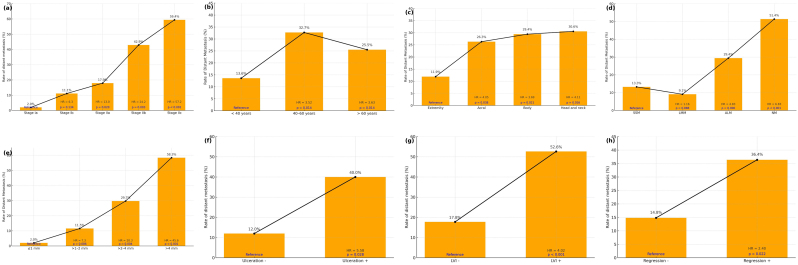
Rates of distant metastasis and HR values observed in patients according to melanoma stage (a), age groups (b), melanoma localization (c), histological subtype (d), Breslow thickness (e), ulceration (f), lymphovascular invasion (g), and regression (h).

Although not statistically significant in the overall analysis, the univariate analysis indicated that melanomas on the extremities were associated with a lower risk of metastasis compared to those in other anatomical sites (p = 0.011, HR = 0.26 [0.09–0.73]). Regarding melanoma histological subtypes, lentigo maligna melanoma (LMM) and superficial spreading melanoma (SSM) exhibited similar characteristics, whereas nodular melanoma (NM) and acral lentiginous melanoma (ALM) were associated with significantly higher rates of distant metastasis. However, the melanoma histological subtype did not retain its significance in multivariate analysis. The relationship between clinical, demographic, and histopathological variables and distant metastasis is presented in Fig. 2, and the univariate and multivariate regression analyses are presented in Table 3.

**Table 3:** Regression analysis of the relationship between clinical, demographic, and histopathological variables and distant metastasis.

	Univariate				Multivariate			
	p	HR	95% CI	χ^2^ (Wald)	p	HR	95% CI	χ^2^ (Wald)
**Age at diagnosis (years, continuous)**	0.015**	1.03	1.01–1.05	5.9	0.050**	1.03	1.00–1.05	3.9
**Age at diagnosis (years)**	0.032**			6.9				
< 40 (reference)		1.00						
40‒60	0.014	3.52	1.29‒9.63	6.0				
> 60	0.014	3.63	1.30‒10.14	6.7				
**Male Gender**	0.321	1.41	0.72‒2.76	1.0				
**Localization**	0.086			6.6				
Extremity (reference)		1.00						
Head and neck	0.016	4.11	1.31‒12.96	5.8				
Body	0.021	3.69	1.22‒11.13	5.3				
Acral	0.038	4.05	1.08‒15.17	4.3				
**Histologic subtype**	< 0.001**			15.0	0.591			1.9
SSM (reference)		1.00				1.00		
NM	<0.001	6.83	2.98–15.66	20.6	0.681	1.23	0.45–3.36	0.2
ALM	0.006	4.83	1.57‒14.89	7.5	0.367	0.49	0.10–2.33	0.8
LMM	0.888	1.16	0.15–9.29	0.0	0.734	1.44	0.18–11.91	0.1
**Breslow (mm, continuous)**	< 0.001**	1.28	1.19‒1.39	39.1	0.008**	1.19	1.05–1.35	7.0
**Breslow (mm)**	< 0.001**			22.2				
≤ 1 (reference)		1.00						
> 1‒2	0.086	7.27	0.75‒70.26	2.9				
> 2‒4	0.004**	20.28	2.61‒157.35	8.3				
> 4	< 0.001**	45.64	6.10‒341.47	13.8				
**Ulceration (present)**	< 0.001**	5.58	2.59–12.04	19.2	0.028**	3.05	1.13–8.20	4.9
**Mitosis (present)**	0.075	29.59	0.71–1233.61	3.2				
**Regression (present)**	0.022**	2.40	1.1–5.08	5.2	0.138	1.88	0.82–4.30	2.2
**LVI (present)**	< 0.001**	4.02	1.86–8.69	12.5	0.002**	4.20	1.67–10.57	9.3
**TIL (present)**	0.434	0.72	0.32–1.64	0.612				

SSM, Superficial Spreading Melanoma; NM, Nodular Melanoma; ALM, Acral Lentiginous Melanoma; LMM, Lentigo Maligna Melanoma; LVI, Lymphovascular Invasion; TIL, Tumor-Infiltrating Lymphocyte.

### Association of distant metastasis with histopathological data

In univariate analysis, Breslow thickness, ulceration, LVI, and regression were significantly associated with distant metastasis. However, in multivariate analysis, LVI (p = 0.002, HR = 4.20 [1.67–10.57]), ulceration (p = 0.028, HR = 3.05 [1.13–8.20]), and Breslow thickness (p = 0.008, HR = 1.19 [1.05–1.35]) remained significant predictors, in order of their predictive value.

**Fig. 3:**
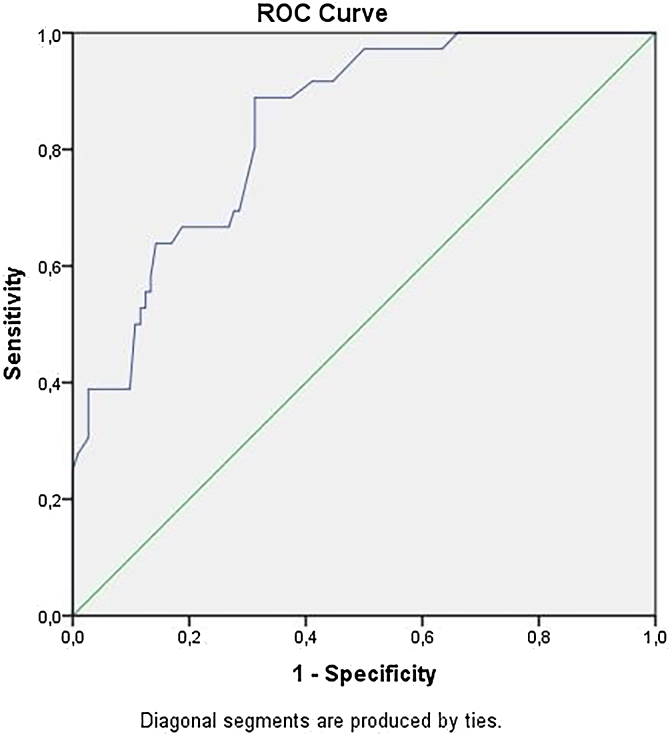
Receiver operating characteristic curve showing the association of Breslow thickness with distant metastasis.

Categorical analysis of Breslow thickness showed a significant difference between the groups (p < 0.001). Detailed analysis revealed that this difference was due to an increased risk of metastasis in individuals with Breslow thickness > 2 mm compared to thinner melanomas (p < 0.001; HR = 11.08 [3.90–31.48]). In the ROC curve analysis, the optimal cut-off value for Breslow thickness in predicting distant metastasis was determined to be 2.73 mm (p < 0.001, AUC = 0.841) (Table 4). Fig. 3 shows the ROC curve of the Breslow thickness.

**Table 4:** Breslow thickness and roc curve analysis for distant metastasis.

Risk Factor	AUC (95% CI)	Cut-Off	p	Sensitivity	Specificity
Breslow thickness	84.1 (0.773‒0.908)	2.7250	< 0.001	0.694	0.714

ROC, Receiver Operating Characteristic, AUC, Area Under the Curve.

### Association of regional LN metastasis with clinical, demographic, and histopathological data

In the univariate analysis, only ulceration (p = 0.033) and Breslow thickness (p = 0.051, borderline significance) emerged as significant predictors of LN metastasis. Additionally, patients with regional LN metastases had a significantly higher risk of developing distant metastasis (p = 0.011, HR = 2.42 [1.23–4.76]).

## Discussion

In this study, the authors investigated the predictive parameters for distant metastasis in early-stage cutaneous melanoma within a cohort of Turkish patients who were followed for metastatic disease progression over 20 years in a dermatology setting at a reference university hospital. The present findings identified increasing age, greater Breslow thickness, ulceration, and LVI as independent risk factors for the development of distant metastases.

The clinicopathological characteristics of cutaneous melanoma in Turkish patients were first analyzed in an oncology clinic by Taş et al. in 2006. In this study including a total of 475 patients across localized, regional, and advanced disease stages, factors such as the nodular histological subtype, greater Breslow thickness, extensive invasion, the presence of ulceration, advanced stage, recurrence, male sex, older age, visceral recurrence, and high mitotic activity were found to be associated with poor OS in localized melanoma.^10^ In subsequent years, findings differed from earlier results, and the authors reported that while LVI was associated with metastatic disease, regression, patient age, tumor localization, and the ALM histological subtype were not.^3^, ^5^, ^9^, ^10^, ^11^, ^12^, ^13^, ^14^ There are also several differences between these findings and those of previous studies. This is likely due to the fact that earlier studies excluded certain melanoma subtypes, such as LMM, and included patients at all disease stages who were followed in oncology clinics. Additionally, prognostic markers such as Breslow thickness, ulceration, regression, and LVI were often evaluated not only in early-stage patients but also in those diagnosed at advanced or metastatic stages.^10^, ^11^, ^12^, ^13^, ^14^, ^15^, ^16^

Regarding studies worldwide, a Brazilian cohort identified male sex, nodular clinical and histologic subtype, Breslow thickness greater than 4 mm, and histologic ulceration as prognostic factors for melanoma metastasis.^17^ Similarly, a Korean study reported that nodular histologic subtype, ulceration, and increased Breslow thickness were significant predictors of metastatic spread in primary cutaneous melanoma.^18^ A Taiwanese study focusing on stage I and II cutaneous melanomas demonstrated that a tumor thickness greater than 4 mm and the presence of ulceration were independent risk factors for hematogenous dissemination.^19^ Another study from Taiwan found that male sex, LVI, and positive LN status were prognostic indicators for both locoregional recurrence and distant metastasis.^20^ In the US, a study reported that among patients with localized and regional disease, the relative risk of melanoma-specific mortality increased with advancing age at diagnosis, with this effect being more pronounced in women than in men.^21^ Finally, a recent meta-analysis investigating clinical and histological factors associated with distant metastasis in melanoma found that male sex, head and neck location (compared to the trunk), NM and LMM subtypes (compared to SSM), increased Breslow thickness, ulceration, and LVI were all significantly associated with a higher risk of distant metastasis. Conversely, the presence of regression was identified as a negative predictor of distant metastatic spread.^9^ The presence of differing results not only in national but also in international studies highlights the importance of conducting research in diverse clinical settings and across different ethnic and geographic populations, in order to enhance the evidence base regarding the progression and prognosis of early-stage melanoma.

In the studied cohort, age was the only independent demographic predictor of distant metastasis. This risk increased significantly, particularly in individuals over the age of 40. Although the highest metastasis rate was observed in the 40–60 age group (32.7%), the > 60 age group had a higher HR (3.63 vs. 3.52), suggesting an earlier onset of metastasis in this population. These findings indicate that advanced age may be associated with a more aggressive disease course or reduced immune surveillance. In the studies by Taş et al., various age thresholds such as < 40, 40–60, ≥ 50, ≥ 60, and ≥ 75-years were used to evaluate metastasis and OS. While some studies found no impact of age on prognosis, others associated older age with poorer outcomes.^3^, ^10^, ^11^, ^12^, ^13^, ^14^, ^15^, ^16^ Numerous studies have shown that tumors in elderly patients exhibit greater Breslow thickness and a higher prevalence of ulceration and LVI, all of which are established prognostic indicators of aggressive disease and a heightened risk of distant metastasis.^6^, ^7^, ^10^ The underlying mechanisms behind the age-related differences in melanoma behavior are not fully understood but may involve a combination of biological and clinical factors. These include the decline in immune system efficiency associated with aging, impairing the body's ability to eliminate malignant cells; cumulative ultraviolet exposure over time, leading to a higher mutational burden and increased genomic instability in melanoma cells; the age-related decrease in melanin, a pigment that can inhibit melanoma cell proliferation; and delays in diagnosis due to reduced access to healthcare or differences in skin surveillance practices in elderly patients.^22^, ^23^, ^24^

Breslow thickness measures the depth of tumor invasion and serves as a reliable indicator of the likelihood of metastatic spread and OS. The role of Breslow thickness in melanoma prognosis has been well-documented in both global and Turkish population studies.^10^, ^17^, ^18^ In the study by Taş et al., a Breslow thickness of > 2 mm was found to be a poor prognostic factor for OS and was also found to predict a shorter time to relapse in stage I–II patients.^5^, ^10^, ^15^ On the other hand, the results of another study by the same authors revealed that Breslow thickness had no impact on relapse-free survival or OS in elderly patients.^3^ In the study population, a Breslow thickness greater than 2 mm was significantly associated with distant metastasis in early-stage melanoma. In the ROC curve analysis, the authors found the optimal cut-off value to be 2.73 mm, with a sensitivity of 69% and a specificity of 71%. Although any cut-off point should be interpreted with caution and clinical decisions in patients with early-stage cutaneous melanoma should ideally be based on multiple selection criteria, the use of long-term follow-up data and the determination of an optimal Breslow cut-off value may serve as an initial reference for identifying patients at higher risk of developing distant metastases or for selecting candidates for adjuvant therapy in clinical practice.

In addition to Breslow depth, ulceration emerged as another critical prognostic factor for the risk of distant metastasis, conferring a 3.05-fold increased risk in the multivariate analysis. It was also identified as a predictor for LN metastasis. The erosion or destruction of the epidermis overlying the tumor generally reflects a more aggressive tumor biology and is indicative of a worse prognosis. Analyses of patients with LN metastases have shown that the absence of ulceration in the primary tumor is associated with a more favorable prognosis. In the literature, ulceration has also been identified as a strong predictor of LN metastasis and holds importance in patient selection for SLNB.^25^ In the eighth edition of the AJCC melanoma staging system, ulceration is considered the only histopathological parameter determining tumor stage apart from Breslow thickness.^2^

In this study, LVI was the strongest independent histopathologic predictor of distant metastasis, demonstrating a 4.20-fold increased risk in the multivariate analysis. Existing evidence suggests that the invasion of melanoma cells into lymphatic or blood vessels provides a pathway for tumor cells to access the circulation, facilitating their dissemination to distant sites and the formation of metastatic tumors.^22^ The definitive and prognostic role of LVI in localized cutaneous melanoma was associated with worse OS in previous studies conducted in Turkey, similar to findings observed in Western countries.^9^, ^23^ In a meta-analysis of histological factors associated with distant metastasis, LVI was identified as a strong risk factor for distant metastasis, with an HR comparable to that of ulceration (2.86 vs. 2.05), suggesting the potential for wider applicability of these results.^9^

Although tumor location was not an independent prognostic factor in the multivariate analysis, it influenced metastatic patterns in the univariate analysis. Melanomas located on the extremities were associated with a significantly lower risk of distant metastasis compared to those on the trunk, head and neck, or acral areas. Previous studies have indicated that extremity melanomas predominantly develop locoregional recurrences, while axial melanomas tend to progress primarily with distant recurrences.^4^ It has been suggested that melanomas arising in chronically sun-exposed areas, such as the extremities, may exhibit increased melanin pigmentation, which is associated with more favorable biological behavior and reduced metastatic potential.^26^ However, this hypothesis does not sufficiently account for the aggressive metastatic risk observed in melanomas of the head and neck, which is also chronically sun-exposed. In the present detailed analysis, head and neck melanomas were identified as the most common subtype and the earliest to develop distant metastases. Among the other subtypes, trunk melanomas showed a higher frequency of metastasis compared to acral melanomas (29.4% vs. 26.3%); however, the HRs suggested that acral melanomas may develop metastases earlier than trunk melanomas (4.05 vs. 3.69). In the studies conducted by Taş et al., tumor localization was generally not found to affect prognosis; however, in one of their studies, they reported that scalp melanomas were associated with poorer survival compared to melanomas at other sites.^10^, ^12^, ^15^ Recent studies have suggested that the anatomical site of the primary melanoma may also play a role in the age-related differences in metastatic risk. Melanomas located on the trunk and lower extremities have been associated with a higher risk of metastatic disease in older patients compared to those located on the head and neck.^27^ Although these findings are conflicting, they highlight the importance of considering anatomical location in risk prediction, and further studies with larger sample sizes are needed to clarify these associations.

The histological subtype of melanoma also affected metastatic patterns in this study, with SSM and LMM associated with a better prognosis compared to NM and ALM. LMM showed the lowest frequency and latest onset of metastasis, whereas NM and ALM exhibited the highest risk and the most rapid progression to metastasis, respectively. The relatively low rate of metastasis observed in LMM may be attributed to its characteristically slow and chronic course of progression. Although the high metastatic potential of NM is well known, the prognostic implications of ALM and plantar melanomas have been variably reported in the literature. Despite their aggressive features, previous studies found no correlation with nodal involvement, recurrence, distant metastasis, or poor survival outcomes.^13^, ^14^ However, a recent meta-analysis identified the histologic subtype of the tumor as a strong predictor of metastasis, with NM, LMM, and ALM all showing an increased likelihood of developing distant metastasis compared to SSM (HR: 2.51, 1.87, and 1.47, respectively). Notably, a reduction in distant metastasis-free survival was found to be associated only with ALM.^9^ It remains uncertain whether these differences reflect true biological variation or are influenced by other factors such as delayed diagnosis and treatment due to anatomical localization, or by the effects of chronic pressure and trauma on tumor behaviour. However, the available evidence suggests that the inflammatory cells and other components within the tumor microenvironment can influence the ability of cancer cells to escape immune surveillance, promote angiogenesis, and facilitate invasion of the vasculature.^13^, ^14^

This study has certain limitations. The retrospective design and reliance on a single-center dataset may limit the generalizability of the findings. Additionally, while the study population represents a Turkish cohort, the relatively small sample size may not fully capture the variability across broader geographic or ethnic populations. On the other side, the strengths of this study include its long-term follow-up period, comprehensive data collection, and rigorous exclusion criteria, which ensured a homogenous cohort of early-stage melanoma patients. To assess the adequacy of the sample for multivariable analysis, a post-hoc power calculation based on observed metastasis rates was performed, and the power exceeded 85% confirming that the sample was sufficient for the study. By including all complete data, the authors aimed to reduce information bias and controlled for confounding effects using multivariate Cox regression analysis. Additionally, to the best of our knowledge, this study is the first to investigate the predictive value of clinical, demographic, and histopathological data on distant metastasis in Turkish patients with early cutaneous melanoma.

In conclusion, although each parameter may hold prognostic significance individually, the present study identified increasing age, greater Breslow thickness, ulceration, and LVI as independent risk factors for distant metastasis in early-stage cutaneous melanoma. Notably, the risk was significantly elevated in individuals over the age of 40 and in those with a Breslow thickness exceeding 2.73 mm. Nonetheless, further studies in this population are warranted to validate these findings, support their integration into clinical practice, and ultimately enhance patient outcomes.

## ORCID ID

Elif Kazaz: 0000-0003-0838-5569

## Data availability statement

The data of the study can be shared upon reasonable request.

## Ethics statement

The authors confirm that the journal's ethical policies, as outlined on the journal's author guidelines page, have been adhered to. The study protocol was approved by the Local Ethics Committee, following the guidelines established by the Declaration of Helsinki. (Reference Number: 2025/03-05, Date: 22.01.2025).

## Financial support

This research received no specific grant from any funding agency in the public, commercial, or not-for-profit sectors.

## Authors’ contributions

Ozlem Ozbagcivan: Approval of the final version of the manuscript, critical literature review, data collection, analysis and interpretation, effective participation in research orientation, intellectual participation in propaedeutic and/or therapeutic management of studied cases, manuscript critical review, preparation and writing of the manuscript, statistical analysis, study conception and planning.

Elif Kazaz: Approval of the final version of the manuscript, data collection, analysis and interpretation, manuscript critical review.

## Research data availability

The entire dataset supporting the results of this study was published in this article.

## Conflicts of interest

The authors declare no commercial or financial conflicts of interest, including those related to the specific brands of materials used in this study.
